# Syntheses of Radioiodinated Pyrimidine-2,4,6-Triones as Potential Agents for Non-Invasive Imaging of Matrix Metalloproteinases

**DOI:** 10.3390/ph10020049

**Published:** 2017-05-30

**Authors:** Hans-Jörg Breyholz, Klaus Kopka, Michael Schäfers, Stefan Wagner

**Affiliations:** 1 Department of Nuclear Medicine, University Hospital Münster, Albert-Schweitzer-Campus 1, Building A1, D-48149 Münster, Germany; hans-joerg.breyholz@ukmuenster.de; 2 Division of Radiopharmaceutical Chemistry, German Cancer Research Center (DKFZ), Im Neuenheimer Feld 280, D-69120 Heidelberg, Germany; k.kopka@dkfz-heidelberg.de; 3 European Institute for Molecular Imaging (EIMI), University of Münster, Waldeyerstraße 15, D-48149 Münster, Germany; michael.schaefers@ukmuenster.de

**Keywords:** pyrimidine-2,4,6-triones, radioiodination, matrix metalloproteinase inhibitor, fluorometric in vitro inhibition assays

## Abstract

Dysregulated expression or activation of matrix metalloproteinases (MMPs) is observed in many kinds of live-threatening diseases. Therefore, MMP imaging for example with radiolabelled MMP inhibitors (MMPIs) potentially represents a valuable tool for clinical diagnostics using non-invasive single photon emission computed tomography (SPECT) or positron emission tomography (PET) imaging. This work includes the organic chemical syntheses and in vitro evaluation of five iodinated barbiturate based MMPIs and the selection of derivative **9** for radiosyntheses of isotopologues [^123^I]**9** potentially useful for MMP SPECT imaging and [^124^I]**9** for MMP PET imaging.

## 1. Introduction

The in vivo molecular imaging of locally upregulated and activated matrix metalloproteinases (MMPs) that are observed in pathologies such as cardiovascular diseases, inflammation or cancer remains a substantive clinical issue [1]. Current targeting strategies for noninvasive imaging of MMPs should not only account for high binding affinity and specificity towards the enzyme but also for drug-target residence time [2], subgroup selectivity, sensitivity, target-to-background ratio as well as in vivo stability [3]. Maybe insufficient consideration of these parameters caused that most of the preclinical studies of the past 20 years with radiolabeled MMPIs were either disappointing or remained at a preliminary stage [4,5]. In addition an inadequate validation of the animal models regarding their level of MMP expression leads to challenging data [6].

Our own approaches towards the development of radiolabelled and fluorescent-dye conjugated MMP tracers focused on two different classes of non-peptidic MMPIs, on the one hand hydroxamate-based inhibitors (i.e., derivatives of CGS 27023A and CGS 25966 [7] with a broad-spectrum inhibitory profile) and, on the other hand, pyrimidine-2,4,6-trione-based inhibitors (i.e., barbiturates, derivatives of RO-2653 [8,9,10,11] with sub-group selectivity for the gelatinases A (MMP-2) and B (MMP-9), neutrophil collagenase (MMP-8) and the membrane-bound MMPs MT-1-MMP (MMP-14) and MT-3-MMP (MMP-16)). Initially, in 2005 we suggested in the latter project a first radiolabelled barbiturate-based MMPI, compound **12** (see Table 2) labelled with the radionuclide iodine-125 (^125^I), for first in vitro and ex vivo applications [12]. In 2008 we developed for the first time the barbiturate-based near-infrared fluorescent photo probe Cy5.5-AF443 that was suitable for in vitro and in vivo imaging of the gelatinases MMP-2 and MMP-9 [13,14]. In 2010 we published the radiosynthesis and evaluation of the first fluorine-18 (^18^F) labelled barbiturate-based MMPI [15] and two years later, of several more hydrophilic radiofluorinated analogues with improved biodistribution behavior [16]. Moreover, a gallium-68 (^68^Ga) labelled version was introduced by our group in 2012 [17]. Favorable MMP binding affinities for our barbiturate-based tracers were indeed measured by in vitro assays and in vivo biodistribution studies using wt-mice. However, in animal models with increased MMP expression mentioned barbiturate-based tracers did not meet the expectations. Anyhow in vivo MMP imaging was feasible and specific with our hitherto most encouraging optical imaging probe Cy5.5-AF443 suggesting the assumption that improved imaging performance of the photoprobe Cy5.5-AF443 compared to the barbiturate radiotracers is caused by the cyanine dye substituent with the four hydrophilic sulfonic acid moieties. Actually, these structural characteristics change the physicochemical properties and accordingly the essential biodistribution pattern influenced i.a. by the excretion routes (renal or hepatobiliary), plasma-protein binding, binding to non-target-organs and/or off-target interactions with other proteins. In summary, adopting or transferring features from optical tracers to radiotracers could support their development, an aspect that was recently reviewed by Faust et al. [18]. Therefore, the aim of this work was the synthesis of radioiodinated barbiturate-based MMPI tracers with increased hydrophilicity for potential SPECT/PET imaging. Radionuclides iodine-123 and iodine-124 were used for the radiosyntheses and applied on our target molecule **9**, which is ca. 3 log units more hydrophilic as compared to our initial preclinical research tracer [^125^I]**12** (see Table 2) [12]. To achieve increased hydrophilicity two different chemical modifications of the C5 phenoxyphenyl moiety in **12** that occupies the S1’ enzyme pocket were realized. Moreover the commercially available radionuclides iodine-123 (for SPECT) and iodine-124 (for PET) exhibit prolonged half-lives t_½_ compared to the most common γ-emitter for SPECT technetium-99m (t_½_ 13.2 h vs. 6.0 h) and β^+^-emitter for PET fluorine-18 (t_½_ 4.2 d vs. 110 min) allowing long-term studies with the corresponding ^123/124^I-labelled barbiturate-based tracer in the next steps.

## 2. Results and Discussion

### 2.1. Chemistry

**Scheme 1:**
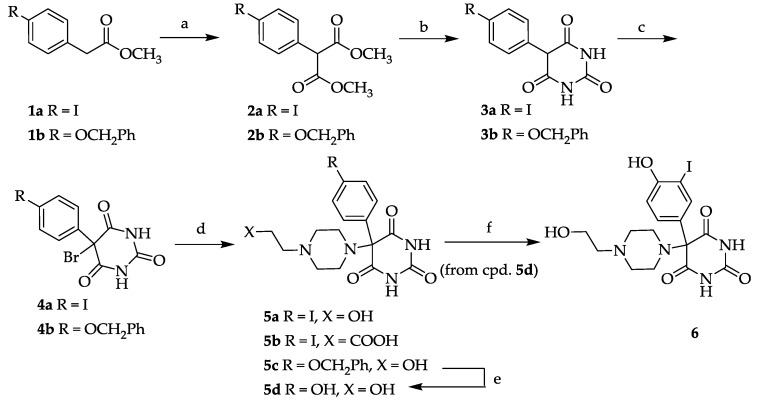
Syntheses of phenyl barbiturates **5a**–**d** and **6**. *Reagents and yields*: (a) NaH, dimethyl carbonate, dioxane, 82% (**2a**), 73% (**2b**); (b) urea, NaOEt, EtOH, 29% (**3a**), 91% (**3b**); (c) NBS, dibenzoyl peroxide, CCl_4_, 51% (**4a**); Br_2_, HBr, H_2_O, 89% (**4b**); (d) *N*-(2-hydroxyethyl)-piperazine or 3-(piperazin-1-yl)-propionic acid, MeOH, 53% (**5a**), 18% (**5b**), 28% (**5c**); (e) H_2_, Pd/C, MeOH, 82% (**5d**); (f) NaI, NaOCl, NaOH, MeOH, 12% (**6**).

Phenyl barbiturates **5a**–**d** and **6** were prepared as outlined in Scheme 1 by four- (compounds **5a**–**c**), five- (compound **5d**) or six-step (compound **6**) sequences, respectively.

In detail, 4-iodophenyl acetic acid methylester (**1a**), synthesized by the esterification of commercial 4-iodophenyl acetic acid using MeOH/H_2_SO_4_ was methoxycarbonylated to give the corresponding malonic ester **2a**. 4-Benzyloxyphenyl malonic acid dimethyl ester (**2b**) [19] was obtained from methyl 2-((4-benzyloxy)phenyl)acetate (**1b**) [20] analogous to **2a** by the literature procedure. Malonic esters **2a** and **2b** were cyclized with urea using sodium ethoxide as a base to yield the 5-phenylbarbituric acid derivatives **3a** and **3b**, which subsequently were brominated with *N*-bromosuccinimide (NBS) (compound **3a**) or bromine/HBr (compound **3b**), resulting in the 5-bromo-5-phenyl barbituric acids **4a** and **4b**. These were reacted with either of the commercially available piperazines *N*-(2-hydroxyethyl)-piperazine or 3-(piperazin-1-yl)-propionic acid in MeOH to yield the barbituric acid derivatives **5a**–**c** in overall yields of 6% (**5a**), 2% (**5b**) and 17% (**5c**). Cleavage of the benzyloxy group in **5c** was achieved by catalytic hydrogenation (H_2_, Pd/C, MeOH) resulting in the hydroxyl compound **5d** in 14% overall yield. Iodination of **5d** using sodium iodide, sodium hypochlorite and sodium hydroxide in methanol yielded the *ortho* iodination product **6** in 2% overall yield.

**Scheme 2:**
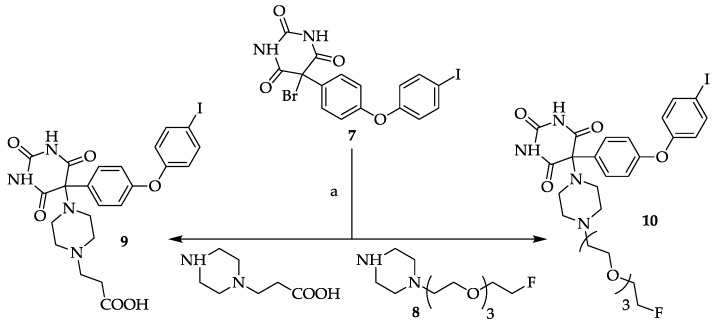
Syntheses of compounds **9** and **10**. *Reagents and yields*: (a) MeOH, 83% (**9**), 8% (**10**).

To obtain carboxy derivative **9** and fluoro derivative **10** (Scheme 2) the bromo intermediate **7**, whose synthesis was described previously [12], was reacted either with commercially available 3-(piperazin-1-yl)-propionic acid or piperazine **8** [15]. The yields were 83% for **9** and 8% for **10** (Scheme 2).

### 2.2. Enzyme Assays and clogD Values

The MMP inhibition potencies of the barbituric acid derivatives **5a**, **5b**, **6**, **9** and **10** were measured in fluorometric in vitro inhibition assays as described previously [21]. The IC_50_-values of **5a**, **5b** and **6** were determined for gelatinases MMP-2 and MMP-9 (Table 1), the IC_50_-values of **9** and **10** for MMP-2, MMP-8, MMP-9, MMP-13 and MMP-14 (only **9**) (Table 2). The results are depicted in Table 1 and Table 2. The tables also contain the calculated logP/logD values (clogP/clogD) of the synthesized barbituric acids derivatives to indicate the changes of lipophilicities caused by the structural modifications.

Replacement of the phenoxyphenyl moiety in **12** (see Table 1) by a phenyl group resulted in compounds **5a**–**d** and **6**. As expected the lipophilicities of iodinated **5a**–**b** and **6** are significantly reduced, with clogD values ranging between −1.34 and 1.26 compared to **12** with a clogD value of 3.53. On the other hand the IC_50_ values for MMP-2 and -9 of these compounds are generally increased compared to the derivatives with a phenoxyphenyl residue (Table 2). This is also expected because the phenoxyphenyl core is optimized for the deep and narrow S_1_’ pocket of the target MMPs [8]. While **5b** and **6** possess IC_50_ values for MMP-2 and -9 in the micromolar range (0.67–1.6 µM, Table 1), **5a** is at least a potent MMP-2 inhibitor with an IC_50_ value of 10 nM.

The second hydrophilic modification included the substitution of the hydroxy group with a carboxy group in the piperazine residue of **12** to yield carboxylic acid **9** resulting in a clogD shift of 2.6 units (approximately 400 fold increased water solubility, Table 2) from 3.53 towards 0.92. Despite this modification the high MMP-2- and -9-inhibition potency of **12** was only marginally influenced (Table 2, **12**: IC_50_ (MMP-2) = 7 nM, IC_50_ (MMP-9) = 2 nM. **9**: IC_50_ (MMP-2) = 29 nM, IC_50_ (MMP-9) = 1.3 nM). Moreover, in vitro data showed, that **9** was also a nanomolar inhibitor of collagenase 3 (MMP-13) and MMP-14 (MT-1 MMP), but only a micromolar inhibitor of neutrophil collagenase (MMP-8) (**9**: (IC_50_ (MMP-14) = 49 nM (Table 2, footnote b), IC_50_ (MMP-8) = 1170 nM, IC_50_ (MMP-13) = 362 nM). In summary, compound **9** confirms the results from Grams et al. [8] that 5,5-disubstituted barbiturates with a para-substituted phenoxyphenyl unit represent potent inhibitors for MMP-2, -9 and -14. Additionally, as shown by comparison of the IC_50_ values of **9** and **13** with **10** the elongation of the substituent of the piperazine residue with fluorinated tri-ethylenglycol resulted in a decrease of selectivity for unknown reasons. From this selection of iodinated barbiturate derivatives carboxylic acid **9** was chosen for radiochemical synthesis of the radioiodinated isotopologues [^123/124^I]**9** (see Section 2.3) because this compound possesses the most favourable characteristics indicated by clogD and IC_50_-values.

### 2.3 Radiochemistry

**Scheme 3:**
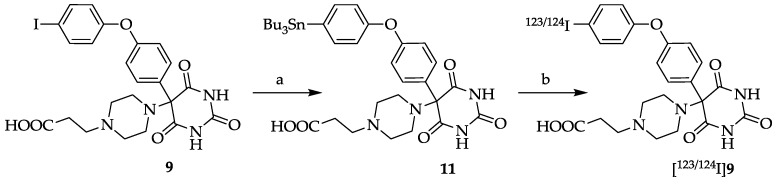
Precursor synthesis of **11** and radiosynthesis of the barbituric acid-based MMP-targeted model tracer [^123/124^I]**9**. *Reagents and yields*: (a) Bu_3_SnH or Bu_6_Sn_2_ (2.0 eq.), PdCl_2_(PMePh_2_)_2_ or PdCl_2_ (3 mol%), KOAc (3.0 eq.), *N*-methyl-pyrrolidone, 28%; (b) [^123/124^I]NaI, chloroamine-T hydrate, 0.1 M K_2_HPO_4_.

Palladium-catalyzed cross-coupling reaction (Stille coupling) of non-radioactive reference compound **9** with tributyltin hydride (or hexabutylditin) (yield: 28%) [20] provided the stannyl precursor **11** (Scheme 3).

Subsequent radioiododestannylation of **11** with no-carrier-added (n. c. a.) [^123/124^I]NaI and chloroamine-T led to the radioligands [^123/124^I]**9**. The decay-corrected radiochemical yields were 28 ± 7% (*n* = 6) for [^123^I]**9** and 44 ± 6% (*n* = 3) for [^124^I]**9** at the end of synthesis (EOS) and the radiochemical purities were 95 ± 3% for [^123^I]**9** and 93 ± 5% for [^124^I]**9** (as determined by radio-HPLC). The molar activities are 0.2–6.3 GBq/μmol and 0.4–14.0 GBq/μmol, respectively. The identities of [^123/124^I]**9** were proven by HPLC (coinjection and coelution with reference compound **9**).

## 3. Materials and Methods

### 3.1. General Methods. Chemistry

All chemicals, reagents and solvents for the synthesis of the compounds were of analytical grade, purchased from commercial sources and used without further purification, unless otherwise specified. Melting points were determined in capillary tubes on a SMP3 capillary melting point apparatus (Stuart Scientific, Staffordshire, UK) and are uncorrected. ^1^H-NMR, ^13^C-NMR and ^19^F-NMR spectra were recorded on ARX 300 and/or AMX 400 spectrometers (Bruker, Karlsruhe, Germany). CDCl_3_ contained tetramethylsilane (TMS) as an internal standard. Mass spectra were obtained on a MAT 212 (EI = 70 eV) spectrometer (Varian Medical Systems, Palo Alto, CA, USA) and a Bruker MALDI-TOF-MS Reflex IV instrument (matrix: DHB). Exact mass analyses were conducted on a Quattro LC (Waters, Milford, MA, USA) and a Bruker MicroTof apparatus. Elemental analyses were realized by a Vario EL III analyzer (Elementar Analysensysteme Comp., Hanau, Germany). All aforementioned spectroscopic and analytical investigations were done by staff members of the Institute of Organic Chemistry, University of Münster, Germany. All purifications of compounds and determinations of purity by HPLC were performed by using a gradient RP-HPLC system (Knauer, Berlin, Germany) equipped with two K-1800 pumps, an S-2500 UV detector and RP-HPLC Nucleosil Eurosphere 100-10 C-18 columns for analytical (250 mm × 4.6 mm) purposes. The following eluents were used (unless specified otherwise): eluent A: water (0.1% TFA), eluent B: acetonitrile (0.1% TFA). The following conditions were used (unless specified otherwise): Gradient from 90% A to 20% A over 30 min, constant 20% A over 5 min and from 20% A to 90% A over 5 min, at a flow rate of 1.5 mL/min, detection at λ = 254 nm.

### 3.2. General Procedure for 4-Iodophenyl Malonic Acid Dimethyl Ester *(**2a**)* and 4-Benzyloxyphenyl Malonic Acid Dimethyl Ester *(**2b**)*

A suspension of 4.00 g NaH (166 mmol, 6.66 g of a 60% suspension in paraffin; the paraffin was removed by repeated washings with petroleum benzene) and 48.0 g (533 mmol) dimethyl carbonate in 80 mL absolute dioxane was heated to 100–120 °C and a solution of 23.00 g (83.3 mmol) 4-Iodophenyl acetic acid methylester (**1a**, prepared by the esterification of 4-iodophenyl acetic acid using MeOH/H_2_SO_4_) in absolute dioxane (125 mL) was added dropwise over a period of 1 h. Refluxing was continued for 3 h and the reaction mixture was allowed to come to room temperature overnight. The mixture was poured onto ice water and subsequently extracted with methylene chloride (3 ×). The combined organic layers were washed with water (1 ×), brine (1 ×), dried (Na_2_SO_4_) and concentrated. The crude **2a** was used in the next step without further purification. Yield: 22.8 g (68.2 mmol, 82%). ^1^H-NMR (300 MHz, DMSO-*d_6_*): δ [ppm]: 7.90 (d, ^3^*J* = 8.3 Hz, 2 H, H_Aryl_), 7.35 (d, ^3^*J* = 8.3 Hz, 2 H, H_Aryl_), 5.18 (s, 1 H, CH), 3.83 (s, 6 H, CH_3_). ^13^C-NMR (75.5 MHz, DMSO-*d_6_*): δ [ppm]: 168.46, 138.14, 133.10, 131.88, 94.86, 55.99, 53.11. 4-Benzyloxyphenyl malonic acid dimethyl ester (**2b**) [19] was prepared from methyl 2-((4-benzyloxy)phenyl)acetate **1b** [20] analogous to **2a**.

### 3.3. General Procedure for 5-(4-Iodophenyl)-pyrimidine-2,4,6-trione *(**3a**)* and 5-(4-Benzyloxyphenyl)-pyrimidine-2,4,6-trione *(**3b**)*

Under an argon atmosphere 2 eq. of sodium were dissolved in ethanol (0.35 mL/mmol Na) and 1.7 eq. of urea were added. A solution of malonic ester **2a** or **2b** in ethanol (2.2 mL/mmol) was added dropwise and the reaction mixture was heated to reflux for 6 h. After cooling to room temperature, the mixture was poured onto ice water and adjusted to pH 2 using dilute hydrochloric acid. The precipitate was collected by suction and dried *in vacuo*. **3a**: Yield: 29%. ^1^H-NMR (300 MHz, DMSO-*d_6_*): δ [ppm]: 11.34 (broad, s), 7.68 (broad, d, 2 H, H_Aryl_), 7.09 (broad, d, 2 H, H_Aryl_), 4.92 (s, 1 H, CH). ^13^C-NMR (75.5 MHz, DMSO-*d_6_*): δ [ppm]: 187.77 (C-4/6 enol form), 169.73 (C-4/6 keto form), 151.31 (C-2), 137.41, 132.46, 128 .89, 93.48, 92.09 (C-5 enol form), 54.60 (C-5 keto form). Anal. Calcd for C_10_H_7_IN_2_O_4_: C 36.39, H 2.14, N 8.49. Found: C 36.61, H 2.29, N 8.23. **3b**: Yield: 91%. Mp 233–236 °C. ^1^H-NMR (300 MHz, DMSO-*d_6_*): δ [ppm]: 11.34 (broad, s, OH), 7.4–6.94 (m, 9 H, H_Aryl_), 5.10 (s, 1 H, CH). ^13^C-NMR (75.5 MHz, DMSO-*d_6_*): δ [ppm]: 187.75 (C-4/6 enol form), 169.71 (C-4/6 keto form), 158.25, 151.29 (C-2), 137.39, 132.44, 130.64, 128.14, 115.31, 114.36, 92.06 (C-5 enol form), 69.58, 54.59 (C-5 keto form). Anal. Calcd for C_10_H_14_IN_2_O_4_·H_2_O: C 62.19, H 4.91, N 8.53. Found: C 62.38, H 4.56, N 8.14.

### 3.4. 5-Bromo-5-(4-iodophenyl)-pryrimidine-2,4,6-trione *(**4a**)*

A suspension of **3a** (6.51 g, 19.7 mmol), *N*-bromosuccinimide (4.20 g, 23.6 mmol, 1.2 eq.) and a catalytic amount of dibenzoylperoxide in carbon tetrachloride (400 mL) was heated to reflux for a period of 3 h. After cooling to room temperature the mixture was concentrated, the residue was treated with water and extracted with ethyl acetate (3×). The combined extracts were washed with brine, dried (Na_2_SO_4_) and the solvent was evaporated. The residue was stirred in CHCl_3_ for 2 h to give a colorless solid. Yield: 4.10 g (10.0 mmol, 51%). Mp 183–186 °C. ^1^H-NMR (300 MHz, DMSO-*d_6_*): δ [ppm]: 11.47 (broad, s), 10.94 (broad, s), 8.64 (broad, s), 7.71 (d, 2 H, H_Aryl_), 7.15 (d, 2 H, H_Aryl_). ^13^C-NMR (75.5 MHz, DMSO-*d_6_*): δ [ppm]: 179.66, 170.80, 137.81, 133.18, 127.65, 95.76, 75.99. MS (EI): *m/e* (intensity %): 410 (M^+^, 15), 408 (M^+^, 15), 329 (100), 282 (45), 244 (45), 196 (62), 129 (29), 89 (38), 43 (39). Anal. Calcd for C_10_H_6_BrIN_2_O_3_: C 29.37, H 1.48, N 6.85. Found: C 29.96, H 1.48, N 6.80.

### 3.5. 5-(4-Benzyloxyphenyl)-5-bromo-pryrimidine-2,4,6-trione *(**4b**)*

A suspension of the **3b** (5.00 g, 16.1 mmol) in water (48 mL) was cooled to 0–5 °C and 48% HBr (3.25 mL, 28.4 mmol) and bromine (1.32 mL, 25.8 mmol) were added dropwise. After stirring for 4–5 h at 0–10 °C the precipitate was collected by filtration and dried *in vacuo.* Yield: 5.59 g (14.4 mmol, 89%). Mp 145–147 °C. ^1^H-NMR (300 MHz, DMSO-*d_6_*): δ [ppm]: 11.44 (broad, s), 7.43–7.01 (m, 9 H, H_Aryl_), 5.08 (s, 2 H, CH_2_). ^13^C-NMR (75.5 MHz, DMSO-*d_6_*): δ [ppm]: 171.34, 159.00, 150.21, 137.18, 130.86, 128.79, 128.03, 127.99, 126.88, 115.30, 76.28, 69.70. MS (ESI-EM) *m/e*: 410.9951 (M + Na)^+^ calcd for C_17_H_13_BrN_2_O_4_Na 410.9956. Anal. Calcd for C_17_H_13_BrN_2_O_4_·0.3 H_2_O: C 52.10, H 3.42, N 7.15. Found: C 51.79, H 3.12, N 6.96.

### 3.6. General Procedure for Compounds ***5a***–***5c***

A solution of **4a** or **4b** in methanol (2–4 mL/mmol) was treated with 2 eq. of *N*-(2-hydroxyethyl)-piperazine (in case of **5a** and **5c**) or 3-(piperazin-1-yl)-propionic acid (in case of **5b**) and stirred for 2 d at room temperature. The colorless precipitate was collected by suction and dried *in vacuo.*

#### 3.6.1. *5-[4-(2-Hydroxyethyl)piperazin-1-yl]-5-(4-iodophenyl)pryrimidine-2,4,6-trione* (**5a**)

Yield: 53%. Mp 168–172 °C. ^1^H-NMR (400 MHz, DMSO-*d_6_*): δ [ppm]: 7.72 (d, ^3^*J* = 8.7 Hz, 2 H, H_Aryl_), 7.16 (d, ^3^*J =* 8.7 Hz, 2 H, H_Aryl_), 3.43–2.31 (m, 12 H, CH_2_). ^13^C-NMR (75.5 MHz, DMSO-*d_6_*): δ [ppm]: 169.36, 151.07, 138.72, 131.17, 130.13, 95.72, 85.71, 59.68, 58.20, 53.59, 47.24. MS (ESI-EM) *m/e*: 459.0518 (M + H)^+^ calcd for C_16_H_20_IN_4_O_4_ 459.0524. Anal. Calcd for C_16_H_19_IN_4_O_4_·H_2_O: C 40.35, H 4.23, N 11.76. Found: C 40.93, H 4.54, N 11.47.

#### 3.6.2. *5-[4-(2-Carboxyethyl)piperazin-1-yl]-5-(4-iodophenyl)pyrimidine-2,4,6-trione* (**5b**)

Yield: 18%. Mp 139–142 °C. ^1^H-NMR (300 MHz, DMSO-*d_6_*): δ [ppm]: 9.42 (broad, s), 8.00 (d, ^3^*J* = 8.7 Hz, 2 H, H_Aryl_), 7.61 (d, ^3^*J* = 8.7 Hz, 2 H, H_Aryl_), 3.30–3.21 (m, 4 H, CH_2_), 2.85–2.59 (m, 8 H, CH_2_). ^13^C-NMR (75.5 MHz, DMSO-*d_6_*): δ [ppm]: 173.74, 164.25, 151.49, 138.89, 135.07, 131.70, 86.35, 85.64, 53.23, 49.47, 43.38, 32.01. MS (ESI-EM) *m/e*: 487.0453 (M + H)^+^ calcd for C_17_H_20_IN_4_O_5_ 487.0473. Anal. Calcd for C_17_H_19_IN_4_O_5_·1.8 H_2_O: C 39.88, H 4.04, N 10.80. Found: C 39.59, H 4.23, N 10.48.

#### 3.6.3. *5-[4-(2-Hydroxyethyl)piperazin-1-yl]-5-(4-benzyloxyphenyl)pryrimidine-2,4,6-trione* (**5c**)

Yield: 28%. Mp 211–213 °C. ^1^H-NMR (400 MHz, DMSO-*d_6_*): δ [ppm]: 7.48–7.02 (m, 9 H, H_Aryl_), 5.09 (s, 2 H, CH_2_), 3.48–3.44 (m, 2 H, CH_2_OH), 2.57–2.38 (m, 10 H, CH_2_). ^13^C-NMR (100 MHz, DMSO-*d_6_*): δ [ppm]: 171.08, 159.63, 150.35, 137.67, 130.00, 129.32, 128.78, 128.28, 128.08, 115.76, 74.99, 70.26, 61.07, 59.28, 48.10, 44.91. MS (MALDI-TOF) *m/e*: 461 (M + Na)^+^, 439 (M + H)^+^. Anal. Calcd for C_23_H_26_N_4_O_5_·1 H_2_O: C 60.52, H 6.18, N 12.27. Found: C 60.35, H 5.71, N 12.27.

### 3.7. 5-[4-(2-Hydroxyethyl)piperazin-1-yl]-5-(4-hydroxyphenyl)pryrimidine-2,4,6-trione *(**5d**)*

Compound **5c** (1.66 g, 3.79 mmol) was dissolved in absolute methanol (150 mL), treated with Pd/C (10%, 250 mg) and heated to reflux for 12 h under an H_2_ atmosphere. After cooling to room temperature, the mixture was stirred overnight. The catalyst was filtered off and washed with methanol (80 mL). The solvent was evaporated and the solid residue was dried *in vacuo.* The crude product was taken up in a CHCl_3_/ethylacetate mixture (1/1, v/v) and stirred at room temperature for 2–3 h and finally re-isolated by suction filtration. Yield: 1.08 g (3.11 mmol, 82%). Mp 185 °C. ^1^H-NMR (300 MHz, DMSO-*d_6_*): δ [ppm]: 7.45 (d, ^3^*J* = 8.6 Hz, 2 H, H_Aryl_), 7.03 (d, ^3^*J* = 8.6 Hz, 2 H, H_Aryl_), 3.79 (m, 2 H, CH_2_OH), 2.99–2.69 (m, 10 H, CH_2_). ^13^C-NMR (75.5 MHz, DMSO-*d_6_*): δ [ppm]: 170.66, 158.47, 149.82, 131.22, 129.29, 115.94, 74.63, 59.74, 57.66, 53.73, 46.67. MS (ESI-EM) *m/e*: 349.1521 (M + H)^+^ calcd for C_16_H_21_N_4_O_5_ 349.1506. Anal. Calcd for C_16_H_20_N_4_O_5_·2.2 H_2_O: C 49.56, H 5.77, N 14.44. Found: C 49.08, H 5.54, N 14.02.

### 3.8. 5-(4-Hydroxy-3-iodophenyl)-5-[4-(2-hydroxyethyl)piperazin-1-yl]pryrimidine-2,4,6-trione *(**6**)*

Compound **5d** (500 mg, 1.44 mmol) was dissolved in methanol (10 mL) and treated with 58 mg (1.44 mmol) sodium hydroxide and 216 mg (1.44 mmol) sodium iodide. The solution was cooled in an ice bath and 824 mg (687 µL, 1.44 mmol) sodium hypochlorite (13% active chlorine) were added dropwise over a period of 60 min. The orange suspension was stirred in the ice bath for further 2 h until a nearly colorless solution had formed. The ice bath was removed and 2–3 crystals sodium thiosulfate were added at room temperature. The solution was acidified to pH 6.8 by adding 1 M HCl and extracted with ethylacetate (3 × 30 mL). The combined extracts were washed with brine (1 × 30 mL), dried (MgSO_4_) and evaporated to dryness. Yield: 80 mg (0.17 mmol; 12%). Mp 168–170 °C (decomposition). ^1^H-NMR (300 MHz, DMSO-*d_6_*): δ [ppm]: 11.50 (s, broad, 2 H), 7.60–6.69 (m, 3 H, H_Aryl_), 4.34 (s, 1 H, OH) 3.40–2.28 (m, 12 H, CH_2_). ^13^C-NMR (75.5 MHz, DMSO-*d_6_*): δ [ppm]: 169.87, 157.32, 149.35, 137.99, 129.10, 127.04, 115.37, 84.56, 73.25, 59.71, 58.27, 53.70, 47.11. MS (ESI-EM) *m/e*: 475.0467 (M + H)^+^ calcd for C_16_H_20_IN_4_O_5_ 475.0473. Anal. Calcd for C_16_H_19_IN_4_O_5_·H_2_O: C 39.04, H 4.30, N 11.28. Found: C 39.47, H 4.23, N 11.21.

### 3.9. General Procedure for Compounds ***9*** and ***10***

A solution of 5-bromo-5-[4-(4-iodo-phenoxy)-phenyl]pyrimidine-2,4,6-trione **7** [8,12] in methanol (ca. 5–10 mL/mmol) was treated with 2.0 eq. of the appropriate piperazine (**8** or 3-(piperazin-1-yl)-propionic acid) and stirred at room temperature overnight. The colorless solids which precipitated after ca. 1 h were collected by suction and dried *in vacuo*. Compound **10** was further purified by silica gel column chromatography (EtOAc/MeOH (4/1, v/v) + 1% NEt_3_).

#### 3.9.1. *5-[4-(2-Carboxyethyl)piperazin-1-yl]-5-[4-(4-iodophenoxy)phenyl]pyrimidine-2,4,6-trione* (**9**)

Yield: 83%. Mp: 236–242 °C (decomposition). ^1^H-NMR (400 MHz, DMSO-*d_6_*): δ [ppm]: 9.31 (s, broad, 2 H), 7.95 (d, ^3^*J* = 8.6 Hz, 2 H, H_Aryl_), 7.72 (d, ^3^*J* = 9.0 Hz, 2 H, H_Aryl_), 6.92 (d, ^2^*J* = 9.0 Hz, 2 H, H_Aryl_), 6.87 (d, ^3^*J* = 8.6 Hz, 2 H, H_Aryl_), 3.16–3.09 (m, 2 H, CH_2_), 2.73–2.58 (m, 8 H, CH_2_), 2.51–2.44 (m, 2 H, CH_2_). ^13^C-NMR (100 MHz, DMSO-*d_6_*): δ [ppm]: 173.80, 164.31, 158.77, 151.62, 150.29, 138.66, 135.36, 131.05, 120.00, 118.05, 86.65, 85.42, 53.24, 49.46, 43.32, 40.53. MS (ESI-EM) *m/e*: 420.9687 (M − C_7_H_13_N_2_O_2_)^+^ calcd for C_16_H_10_IN_2_O_4_ 420.9685. Anal. Calcd for C_23_H_23_IN_4_O_6_·2 H_2_O: C 44.96, H 4.43, N 9.12. Found: C 45.16, H 4.70, N 9.52.

#### 3.9.2. *5-(4-(2-(2-(2-(2-Fluoroethoxy)ethoxy)ethoxy)ethyl)piperazin-1-yl)-5-(4-(4-iodophenoxy)phenyl)-pyrimidine-2,4,6-trione* (**10**)

Yield: 8%. Mp 176-177 °C. ^1^H-NMR (300 MHz, DMSO-*d_6_*) δ [ppm]: 9.28 (s, 2 H), 7.84 (d*,* ^3^*J* = 7.8 Hz, 2 H, H_Aryl_), 7.64 (d, ^3^*J* = 7.8 Hz, 2 H, H_Aryl_), 6.81 (d, ^3^*J* = 6.8 Hz, 2 H, H_Aryl_), 6.76 (d, ^3^*J* = 6.8 Hz, 2 H, H_Aryl_), 4.51 (dt, ^2^*J*_H,F_ = 48.1 Hz, 2 H, CH_2_F), 3.56–2.50 (m, 22 H, CH_2_). ^13^C-NMR (75.5 MHz, DMSO-*d_6_*): δ [ppm]: 169.93, 158.40, 151.31, 149.89, 138.31, 135.00, 130.71, 119.61 117.75, 86.35, 85.13, 83.11 (d,|^1^*J*_C,F_| = 165.7 Hz), 70.54, 70.39, 70.12, 69.76, 69.57, 68.15, 56.71, 49.62, 42.98. ^19^F-NMR (282 MHz, CDCl_3_): δ [ppm]: −216.61. MS (ESI-EM) *m/e*: 685.1518 (M + H)^+^ calcd for C_28_H_35_FIN_4_O_7_ 685.1529. The purity of **10** was determined by analytical gradient HPLC to be > 95% (system and conditions see Section 3.1), t*_R_* = 25.92 ± 0.36 min (*n* = 3).

### 3.10. 5-[4-(2-Carboxyethyl)piperazin-1-yl]-5-[4-(4-(tributylstannyl)phenoxy)phenyl]pyrimidine-2,4,6- trione *(**11**)*

PdCl_2_ (PMePh_2_)_2_ (18 mg, 30 µmol, 3 mol%) or PdCl_2_ (18 mg, 100 µmol, 10 mol%), KOAc (294 mg, 3.00 mmol, previously dried for several hours at 100 °C *in vacuo* before use) and *N*-methyl-pyrrolidinone (NMP, 15 mL) were mixed under an argon atmosphere [20]. Compound **9** (578 mg, 1.00 mmol) and tributyltin hydride (582 mg, 2.00 mmol) or hexabutylditin (870 mg, 1.50 mmol) were added and the mixture was heated to 110 °C for 10–15 h. The progress of the reaction was monitored by HPLC (system and conditions see Section 3.1). The retention times t*_R_* were: t*_R_* (**9**): 34.15 ± 1.38 min (*n* = 10), t*_R_* (**11**): 40.83 ± 1.17 min (*n* = 6). When the conversion was complete, the hot black reaction mixture was filtered through a pad of Celite by suction and the filter cake was washed with a small amount of NMP. The orange filtrate was stored at −30 °C overnight. The beige solid which precipitated upon cooling was isolated by suction. When there was no precipitation, the volatile components of the filtrate were removed by short-path distillation. The solid residue was taken up in hot methanol and insoluble components were filtered off. The solvent was removed *in vacuo* and the residue was treated with acetone. Yield: 208 mg (0.28 mmol, 28%). The purity of the product was >95% as determined by analytical gradient HPLC. Mp: 285–286 °C (decomposition). ^1^H-NMR (400 MHz, DMSO-*d_6_*): δ [ppm]: 7.89–7.12 (m, 8 H, H_Aryl_), 3.56–1.94 (m, 12 H, CH_2_), 1.77–1.67 (m, 6 H, CH_2_), 1.56–1.44 (m, 6 H, CH_2_), 1.25–1.20 (m, 6 H, CH_2_), 1.06 (t, ^3^*J* = 8.3 Hz, 9 H, CH_3_). MS (ESI-EM) *m/e*: 585.1847 (M − C_7_H_13_N_2_O_2_)^+^ calcd for C_28_H_37_N_2_O_4_Sn 585.1775. 

### 3.11. General Methods. Radiochemistry

N. c. a [^124^I]NaI was provided by Department of Nuclear Medicine, University Hospital Essen, University Duisburg-Essen, Germany. Typical radioactivities used for the radioiodination were 134 ± 48 MBq [^124^I]NaI in 50 ± 18 μL in 0.01 N NaOH. N. c. a. [^123^I]NaI was purchased from GE Healthcare Buchler GmbH & Co KG (Braunschweig, Germany). Typical radioactivities used for the radioiodination were 136 ± 63 MBq [^123^I]NaI in 10 ± 3 μL in 0.05 N NaOH. Separation, purification and analyses of the radiochemical yields and the radiochemical purities of all radioiodinated compounds were performed by a gradient radio-HPLC chromatograph system (*HPLC A*) using a Knauer K-500 and a Latek P 402 pump, a Knauer K-2000 UV-detector (λ = 254 nm) a Crismatec NaI(Tl) Scintibloc 51 SP51 γ-detector and a RP-HPLC Nucleosil column 100-5 C-18 (250 mm × 4.6 mm), a corresponding precolumn (20 mm × 4.0 mm). Sample injection was carried out using a Rheodyne injector block (type 7125 incl. 200 µL loop). The recorded data were processed by the NINA version 4.9 software (GE Medical Systems-Functional Imaging GmbH, Münster, Germany). Separation and purification of the radiosynthesized compounds were—unless specified otherwise—performed by radio-HPLC (*HPLC A*, see above) using a Nucleosil 100-10 C18 column (250 mm × 8 mm). The recorded data were processed by the NINA version 4.9 software . The radiochemical purities and the specific activities were determined using a radio-HPLC system (*HPLC B*) composed of a Sykam S1021 pump, a Knauer K-2501 UV-detector (λ = 254 nm), a Crismatec NaI(Tl) Scintibloc 51 SP51 γ-detector, a Nucleosil 100-3 C18 column (200 mm × 3 mm), a VICI injector block (type C1 incl. 20 μL loop) and the GINA Star version 4.07 radiochromatography software (raytest Isotopenmeßgeräte GmbH, Straubenhardt, Germany). Radio-TLC was analyzed using a miniGITA TLC-Scanner (raytest Isotopenmeßgeräte GmbH, Straubenhardt, Germany).

### 3.12. 5-[4-(2-Carboxyethyl)piperazin-1-yl]-5-[4-(4-[^123/124^I]-iodophenoxy)phenyl]pyrimidine-2,4,6-trione ([^123/124^I]***9***)

In a conical glass vial 89 µg (0.12 μmol) stannyl precursor **11** in 39 μL MeOH was added to n. c. a. [^123^I]NaI or [^124^I]NaI. The radiosynthesis was started by addition of 34 mg (0.14 μmol) chloroamine-T hydrate in 39 μL 0.1 M K_2_HPO_4_ buffer (pH 7.34). The mixture was vortexed for 10 s and allowed to stand for 5 min at room temperature. To quench the reaction 50 μL of a 10% Na_2_S_2_O_5_ solution (in water for injection) was added and the mixture was vortexed again for 10 s. After 10 min the quenched reaction solution was injected onto the HPLC column (see Section 3.11. *HPLC A*) to isolate the fraction of the radiolabelled product [^124^I]**9** (HPLC conditions: eluent A: CH_3_CN/H_2_O/TFA 950/50/1, v/v/v, eluent B: CH_3_CN/H_2_O/TFA 50/950/1, v/v/v; gradient: from 92% B to 40% B within 45 min, 5 min constant at 40% B, and from 40% B to 92% B within 5 min at a flow rate of 2.0 mL/min, λ = 254 nm; t*_R_* (stannyl precursor **11**) = 40.80 ± 0.81 min (*n* = 3), t*_R_* ([^124^I]**9**) = 32.75 ± 2.13 min (*n = 3*)). The product fraction was evaporated to dryness and redissolved in 0.9% NaCl (200 μL). The decay-corrected radiochemical yield (after evaporation) was 28 ± 7% (*n = 6*) for [^123^I]**9** and 44 ± 6% (*n = 3*) for [^124^I]**9**, respectively. An aliquot (50 μL) was taken for analytical radio-HPLC. The radiochemical purities were 95 ± 3% and 93 ± 5% for [^123^I]**9** and [^124^I]**9**, and the molar activities were 0.2–6.3 GBq/μmol and 0.4–14.0 GBq/μmol, respectively. The identity of [^123^I]**9** and [^124^I]**9** was proven by HPLC using the non-radioactive analog **9**.

### 3.13. In Vitro Enzyme Inhibition Assays

The synthetic fluorometric substrate (7-methoxycoumarin-4-yl) acetyl pro-Leu-Gly-Leu-(3-(2,4-dinitrophenyl)-l-2,3-diamino-propionyl)-Ala-Arg-NH_2_ (R & D Systems, Minneapolis, MN, USA) was used to assay activated MMP-2, MMP-8, MMP-9 and MMP-13 as described previously [21]. The inhibitions of human active MMP-2, MMP-8, MMP-9 MMP-13 and MMP-14 (only **9**) by the barbituric acid derivatives **9** and **10**, of human active MMP-2 and MMP-9 by **5a**, **5b** and **6** were assayed by preincubating MMP-2, MMP-3, MMP-8, MMP-9, MMP-13 or MMP-14 (each at 2 nM) and inhibitor compounds at varying concentrations (10 pM–1 mM) in 50 mM Tris·HCl, pH 7.5, containing 0.2 M NaCl, 5 mM CaCl_2_, 20 μM ZnSO_4_ and 0.05% Brij 35 at 37 °C for 30 min. An aliquot of substrate (10 μL of a 50 μM solution) was then added to 90 μL of the preincubated MMP/inhibitor mixture, and the fluorescence was determined at 37 °C by following product release with time. The fluorescence changes were monitored using a Fusion Universal Microplate Analyzer (Packard Bioscience, Boston, MA, USA) with excitation and emission wavelengths set to 330 and 390 nm, respectively. Reaction rates were measured from the initial 10 min of the reaction profile where product release was linear with time and plotted as a function of inhibitor dose. From the resulting inhibition curves, the IC_50_ values for each inhibitor were calculated by non-linear regression analysis, performed using the Grace 5.1.8 software (Linux).

## 4. Conclusions

Starting from the lipophilic preclincal research tracer [^125^I]**12** (clogD 3.53, Table 2), that was already developed by our group [12], we intended to develop a more hydrophilic radioiodinated barbiturate-based MMP-targeted tracer for the potential non-invasive visualization of activated MMPs in vivo. This was achieved by the substitution of the hydroxy group of **12** by a carboxy group yielding derivative **9** with a hydrophilic shift compared to **12** (clogD 0.92 vs. 3.53). Similar to **12** carboxylic acid **9** represents a very potent inhibitor of MMP-2 and -9 (IC_50_ (MMP-2) = 29 nM, IC_50_ (MMP-9) = 1.3 nM). Therefore radioiodinated analogues [^123/124^I]**9** were successfully synthesized for further in vivo evaluations with SPECT and PET.

## Figures and Tables

**Table 1:**
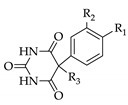
IC_50_ values of phenyl barbiturates **5a**, **5b** and **6**.

Cpd.	R_1_	R_2_	R_3_	IC_50_ [nM]			
				MMP-2	MMP-9	clogP ^a^	clogD ^a^
**5a**	−I	−H	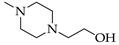	10 ± 3	550 ± 180	1.42	1.26
**5b**	−I	−H	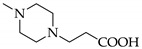	1200 ± 340	1600 ± 600	−0.85	−1.34
**6**	−OH	−I	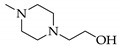	670 ± 210	1300 ± 320	0.75	0.53

^a^ clogP/clogD values were calculated by ACD/Chemsketch version ACD/Labs 6.00 (clogD = clogP at physiological pH (7.4) with consideration of charged species). Values are the mean ± standard deviation (SD) of three assays.

**Table 2:**
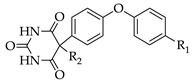
Structures and IC_50_ values of phenoxyphenyl barbiturates **9**–**12**.

Cpd.	R_1_	R_2_	IC_50_ [nM]					
			MMP-2	MMP-8	MMP-9	MMP-13	clogP ^a^	clogD ^a^
**9** ^b^	−I	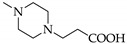	29 ± 2	1170 ± 80	1.3 ± 0.2	362 ± 24	1.40	0.92
**10**	−I	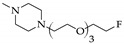	23 ± 5	146 ± 48	17 ± 5	28 ± 11	3.13	2.99
**12**	−I	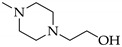	7 ± 1	n. d.	2 ± 0.2	n. d.	3.68	3.53
**13** ^c^	−H	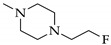	23 ± 9	138 ± 12	7 ± 2	645 ± 17	3.17	2.88 ^d^

^a^ clogP/clogD values were calculated by ACD/Chemsketch version ACD/Labs 6.00 (clogD = clogP at physiological pH (7.4) with consideration of charged species). Values are the mean ± SD of three assays. ^b^ IC_50_ (MMP-14) = 49 ± 8 nM. ^c^ IC_50_ (MMP-1) > 50 µM, IC_50_ (MMP-3) = 760 µM. ^d^ experimental logD = 2.15 ± 0.02. For comparison reasons the IC_50_ values of compound **12** [12] and **13** [15] from previous work are also shown.
